# Physician Assistant/Associate Urology Workforce: A National Analysis

**DOI:** 10.3390/healthcare13030330

**Published:** 2025-02-05

**Authors:** Roderick S. Hooker, Mirela Bruza-Augatis, Kasey Puckett, Andrzej Kozikowski, Todd J. Doran

**Affiliations:** 1 Independent Researcher, 15917 NE Union Rd, Unit 45, Ridgefield, WA 98642, USA; 2 National Commission on Certification of Physician Assistants, 12000 Findley Road, Suite 200, Johns Creek, GA 30097, USA; 3 Physician Assistant Program, National Louis University, 122 S. Michigan Avenue, Chicago, IL 60603, USA

**Keywords:** urology, physician assistant, professional role, healthcare workforce

## Abstract

**Introduction/Background:** The urology workforce is shifting in terms of the number of physicians per capita, age, gender, and availability. To meet this growing need, physician assistants/associates (PAs) and nurse practitioners (NPs) are part of this workforce backfilling effort. However, limited studies have been conducted on the demographic and employment attributes of PAs practicing in urology. Thus, using a national dataset, this study aims to compare the attributes of PAs in the urology workforce compared with PAs in all other surgical and medical disciplines. **Methods:** We analyzed the practice of PAs in urology using data from the 2022 National Commission on Certification of PAs (NCCPA). This study drew on responses from 117,748 board-certified PAs who reported their medical and surgical specialty. Our analysis involved descriptive and inferential statistics, comparing the demographic and practice attributes of PAs in urology (n = 1199) with PAs in all other medical disciplines (n = 116,549). **Results:** In 2022, 1199 (1.0%) PAs were reported to be clinically active in urology. Among PAs in urology, 68.1% self-identified as female, with a median age of 39 [IQR: 32–48]. Compared to PAs in other medical disciplines, PAs in urology resided in urban locations (94.5% vs. 92.5%, *p* = 0.002). They were also more likely to practice in office-based settings (53.6% vs. 37.0%), work over 40 h weekly (37.9% vs. 29.3%), and partake in telemedicine (52.0% vs. 40.1%; all *p* < 0.001). No statistical differences were found among PAs in urology versus PAs in all other medical fields related to job satisfaction (*p* = 0.763), symptoms of burnout (*p* = 0.124), and retirement plans in the next 5 years (*p* = 0.442). **Conclusions:** Given the predicted shortfalls of urologists and their changing demographic composition, this study has important implications for practice in the urology workforce. Our findings can inform workforce planning, recruitment strategies, and organizational policies to support the expansion of PAs in urology and help address shortages in this discipline.

## 1. Introduction

It is projected that, by 2030, 21% of the population of the United States (US) will be over 65 and, by 2060, about one in four persons will be over 65 [1]. Older adults generally require more complex care and are at higher risk for serious and chronic health issues [2], which require multi-specialty care and an adequate healthcare workforce [3]. Urological disorders such as urinary incontinence, urinary tract infections, renal disorders, benign prostatic hypertrophy, and prostate cancer are more common in geriatric individuals [4,5,6,7]; thus, with an increase in the aging population and urological disorders in the US, the demand for urology medical services and procedures will continue to increase [8]. Etzoni and colleagues predicted that, with a growing geriatric population in the US, individuals over 65 will require more (64.8%) urologic procedures over time [9]. Ye et al. determined that, from 1990 to 2019, the global incidence of benign prostatic hypertrophy increased by 119% [7]. Another systematic review study discovered that about a quarter (25.8%) of older women in the US had urinary incontinence, and the prevalence of this disorder has increased globally [5].

In 2022, the American Urology Association (AUA) reported 13,976 practicing urologists in the US, and 87.7% (n = 12,253) are actively working, encompassing both adult and pediatric urologists [10]. The AUA Census (2015–2019) also reported that the median age for urologists was 54, with nearly a third (28.5%) of urologists over 65, indicating a significant portion of urologists near retirement [10]. This demographic trend signals that more urology clinicians are needed: Nam and colleagues forecasted shortages of urologists from 2020 to 2060, and this gap could considerably impact access to urologic care [11]. In their report, physician assistants/associates (PAs) and nurse practitioners [(NPs) were identified as clinicians capable of addressing the gap while expanding access to urological care to mitigate the shortage of urologists [11]. Approximately 82% of urologists work collaboratively with PAs and NPs [11]. McKibben and colleagues projected that, from 2015 to 2035, there will be a 1.2% annual growth rate of urologists and a 2.9% growth rate of PAs and NPs within the discipline of urology [12]. The authors estimated that, with the increased growth rate of PAs and NPs, provider shortfalls in urology are predicted to decrease from 46% to 12% by 2035 [12]. The AUA 2015–2019 census report included the employment of PAs and NPs in urology and concluded their use was essential for improving patient care [13].

The growing demand for urological services, combined with the predicted shortage of urologists and the growth of PAs and NPs working in urology, highlights the need for more research in this workforce. However, prior research on primarily consists of small-sample-size studies that group PAs and NPs together and focus mainly on urological procedures and the utilization of PAs and NPs in urology [13,14,15,16,17]. Yet, no comprehensive national study has been conducted on the number of PAs practicing in urology and their characteristics. With limited information on urology PAs, we aim to examine the employment of PAs in urology, focusing on demographics, geographic distribution, compensation, job satisfaction, and practice setting. The findings from this research can assist in informing employers and workforce planners and provide essential information for predictive modeling to forecast the availability of the urology workforce. Data related to the urology workforce and patterns of care are essential for informing urological care and policy.

## 2. Materials and Methods

### 2.1. Study Design

Data for this cross-sectional exploratory study were obtained from the National Commission on Certification of Physician Assistants (NCCPA)—2022 *PA Professional Profile* dataset. The *PA Professional Profile* was launched in 2012 and housed in a secure portal. It was initially designed using the Health Resource and Services Administration (HRSA) Center for Workforce Studies’ Minimum Data Set [18]. The *PA Professional Profile* collects national data on board-certified PAs’ characteristics, demographics, and work-related information such as income, participation in telemedicine, burnout symptoms, and job plans [19].

### 2.2. Target Population and Sample Characteristics

In 2022, there were 168,318 board certified PAs in the US; of these, 140,796 responded to at least a portion of the profile (83.6% response rate) [19]. Among PAs who completed/updated their profile, 117,748 responded to the clinical specialty question, comprising our target population for this study. The responses were voluntary, and the data were de-identified before the analysis. Administrative data, which included age, gender, US region, and urban–rural setting, were nearly complete, and the remainder of the variables used for this study were self-reported.

### 2.3. Procedure

The data for this research study were obtained from the NCCPA PA Professional Profile. We utilized the 2022 workforce national dataset, which collected responses from all board-certified PAs registered in the NCCPA portal who updated or confirmed their profile information from 2020 to 2022. Responses were examined for consistency and potential errors [19], and two researchers independently analyzed and validated the results. Furthermore, we compared respondents vs. non-respondents using administrative data (99.9% complete data), and no significant differences were identified in terms of gender (*p* = 0.088). However, respondents were slightly older (median age: 38 vs. 36 [non-respondents]), resided in rural–isolated areas (7.2% vs. 5.5%), and were likely to live in the Midwest (19.6% vs. 17.9%) and the South (34.8% vs. 34.4%).

The variables of interest examined in this study included demographics (age, gender, race/ethnicity, US region, and urban–rural setting), practice characteristics (practice setting, years certified as a PA, hours worked weekly, patients seen weekly, secondary position, and participation in telemedicine), and other work-related characteristics (job satisfaction, burnout, plan to leave their principal position in the next 12 months, and plan to retire in the next 5 years) [19].

Age, number of years certified, and patients seen per week are listed as continuous variables. Gender, race, ethnicity, US region, urbanicity, practice setting, hours worked per week, and income range are self-reported categorical variables. For this study, any variables with less than 25 counts were collapsed into smaller categories to protect the anonymity of the participants. For instance, gender is listed as female, male, non-binary, other, or prefer not to answer. Therefore, gender is reported as binary (male and female) due to the small sample size for the other categories. Urbanicity using Rural-Urban Community Area (RUCA) codes [20] were aggregated into four categories (urban, large rural, small rural, and isolated) and then collapsed into urban and rural–isolated categories. Present job satisfaction is reported on a 7-point scale, which was dichotomized as *satisfied* (“completely satisfied”, “mostly satisfied”, and “somewhat satisfied”) and *not satisfied* (“neither satisfied nor dissatisfied”, “somewhat dissatisfied”, and “mostly dissatisfied”) [19]. Burnout was assessed using a validated 5-point scale [21,22] and then collapsed into two categories: *no burnout symptoms* and *one or more burnout symptoms*. The type of practice and clinical setting included 32 different variables, which were categorized into four main sections: office-based, hospital-based, federal government, or other (e.g., rural health clinic, rehabilitation facility, etc.).

### 2.4. Ethical Considerations

This study was determined to be exempt by the Sterling Institutional Review Board (#9942) under the qualification of non-human subject research.

### 2.5. Data Analysis

Data were summarized using descriptive statistics: means and standard deviations (SDs), along with medians and interquartile ranges (IQRs), were computed for continuous variables; frequencies and percentages were calculated for categorical variables. Chi-Square or Mann–Whitney U tests were used to compare the differences between PAs in urology and those in all other medical specialties. Data management and analysis were accomplished with SPSS version 29.0 (IBM Corp, Armonk, NY, USA).

## 3. Results

**Figure 1:**
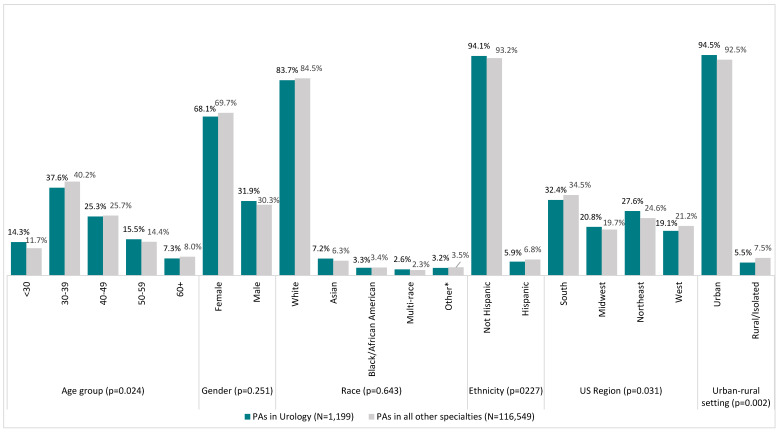
Comparing demographic characteristics of PAs in urology with PAs in all other medical specialties (N = 117,748). * Other includes “other not specified”, “American Indian/Alaska Native”, and “Native Hawaiian/Pacific Islander”. *p*-values were calculated using Pearson Chi-Square analysis.

In 2022, out of 117,748 board-certified PAs, 1.0% (n = 1199) of PAs self-identified as practicing in urology. Figure 1 displays the demographic description of PAs in urology compared to PAs practicing in all other medical disciplines. A slightly higher percentage of PAs in urology reported residing in the Northeast (27.6% vs. 24.6%; *p* = 0.031) and in urban areas (94.5% vs. 92.5%; *p* = 0.002) compared to those in all other medical fields. Both cohorts were akin in age (median [IQR], 39 [32–48] vs. 39 [33–48]; *p* = 0.279), gender (predominately female, *p* = 0.251), race (predominately White, *p* = 0.643), and ethnicity (predominately non-Hispanic/Latino(a), *p* = 0.227).

The findings indicate that PAs in urology are more commonly employed in office-based settings (53.6% vs. 37.0%; *p* < 0.001) and less frequently in hospital settings (36.3% vs. 41.7%; *p* < 0.001) than PAs in all other disciplines. Moreover, compared to their PA colleagues in all other medical disciplines combined, a higher percentage of PAs in urology (all *p* < 0.001) indicated working more than 41–50 h per week (31.1% vs. 23.1%), seeing more patients weekly (median, 65 vs. 60), and participating at a higher percentage rate in telemedicine services (52.0% vs. 40.1%; Table 1).

**Figure 2:**
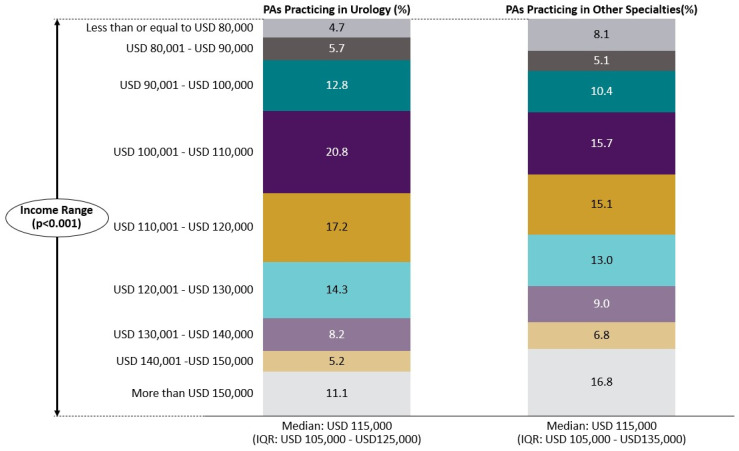
Comparing the 2022 income range distribution of PAs in urology with PAs in all other specialties (N = 117,748). Abbreviation: IQR = interquartile range. *p*-value was calculated using Pearson Chi-Square analysis for income categories.

Interestingly, the median income for both PA cohorts was consistent, USD 115,000, with a higher proportion of urology PAs reporting an income range between USD 80,001 and USD 130,000 (Figure 2).

There were no statistical differences found among PAs in urology vs. those in all other medical disciplines related to job satisfaction (*p* = 0.763), symptoms of burnout (*p* = 0.124), intention to leave their primary clinical role in the next year (*p* = 0.112), or plans to retire in the next five years (*p* = 0.442; Table 2).

## 4. Discussion

Our study highlights essential PA workforce information and complements the findings about PAs in urology from the AUA Census 2015–2019 [23]. We found that, as of 2022, there were 1199 (or 1.0%) clinically active PAs employed in urology. Most PAs in urology self-identified as female, were employed in office-based settings, and typically worked over 40 h per week, and over half (52.0%) partook in telemedicine services. Additionally, PAs in urology reported high levels of job satisfaction and low levels of burnout, and were less likely to indicate retiring in the next 5 years compared to their colleagues in all other medical disciplines.

Our results supplement the AUA reports on PAs in urology in a few other areas [23]. The AUA Urology Care Census from 2015 to 2019 reported that the median age of PAs in urology was 41; the majority (68.2%) were women, 53.4% worked in private practices, and 91.5% practiced in metropolitan areas. Additionally, PAs in urology reported conducting a median of 60 patient visits weekly and working a median of 40 h per week [23]. The same report indicated that in 2018, 25.3% of PAs practicing in urology were experiencing burnout, with 67.3% reporting low levels of emotional exhaustion [23]. Our NCCPA-based study’s strengths include a higher response rate (83.6%) and a large sample size (n = 1199) compared to the 176 PAs who responded to the AUA Census from 2015 to 2019 [23]. When comparing PAs in urology to urologic physicians, some similarities and differences emerged regarding demographics and practice attributes. Similar to PAs in urology, urologists worked predominantly (90%) in urban areas and private practices (51.2%) and worked a median of 55 h per week [4]. Additionally, half of urologists utilize telemedicine to provide services to their patients: 52% of urology PAs do so. In contrast to PAs in urology, urology physicians differ in median age (39 for PAs vs. 54 for physicians) and gender (68.1% female urology PAs vs. 11.6% female urologists) [10]. The finding of a younger PA urology workforce has implications for longer career trajectories; PAs can fill the gap left by retiring urologists [11,13]. Furthermore, the urology workforce is predominantly male, although the proportion of female urologists had increased from 7.7% in 2014 to 11.8% in 2023 [24]. Only 4.5% of urologists specialize in urogynecology/female pelvic medicine and reconstruction [24], which has the highest proportion of female urologists compared to other urology subspecialties [25,26]. The fact that more female PAs practice in urology could assist in filling the gender disparity in the urology workforce. Gender dynamics change the culture of healthcare as female patients may feel more comfortable discussing sensitive urological symptoms with a provider of the same gender, positively influencing the clinician–provider relationship and adherence to treatments. Another distinguishing finding is the burnout rate of PAs practicing in urology compared to urologists. Our study indicated that in 2022, 30.1% of PAs reported one or more burnout symptoms, while the majority (69.9%) did not. This contrasted greatly with burnout among urologic physicians, reported at 48%, which is the highest among all physician specialists [27]. Gupta et al. examined burnout in PAs and NPs using 2019 AUA Census data and found that the burnout rate for PAs was 25.3% (pre-pandemic), and female PAs/NPs had 3.2 times higher odds of burnout compared to males practicing in urology [28]. The authors concluded that more studies need to be undertaken to examine the large differences by gender on burnout for PAs practicing in urology. High job satisfaction for providers is associated with improved retention, productivity, patient satisfaction, healthcare cost, and quality of care [29,30,31,32]. For PAs in particular, high job satisfaction could also improve access to care in times of physician shortage [33]. Hooker and colleagues performed a systematic literature review of job satisfaction among PAs and found that, historically, PAs had high job satisfaction [34]. A few years later, Coplan et al. examined job satisfaction and burnout among a national sample of PAs (n = 15,999) and also determined that job satisfaction was relatively high for PAs [35]. Our study found that 83.2% of PAs in urology indicated being satisfied with their current job, comparable to PAs working in all other medical disciplines (83.6%). Previous studies on PAs practicing in other specialties have reported comparable results regarding job satisfaction. For example, 88% of PAs practicing in oncology [36], 86.6% of PAs in orthopedics [37], 86.0% of PAs in psychiatry [38], 84.9% of PAs in cardiology [39], and 81.8% of PAs in endocrinology [40] indicated being satisfied with their clinical positions, respectively. Work–quality life indicators in the healthcare sector, such as job satisfaction and burnout, are important, especially after the COVID-19 pandemic. PAs have consistently reported high levels of job satisfaction and low levels of burnout [35,41,42], which has implications for workforce planning. However, further research is needed to examine job satisfaction metrics for PAs in urology, considering factors such as gender, age, race, and urbanicity. This research can help recruit and retain PAs from diverse backgrounds, thereby increasing the diversity of the urology workforce to better meet the population’s needs.

The growth rate of PAs in urology is outpacing that of physician urologists. Clinician data from the AUA and NCCPA were compared: between 2014 and 2022, the proportion of physicians specializing in urology increased by 19.4%, while the proportion of PAs practicing primarily in urology rose by 37.3% over the same period [19,43,44]. This trend reflects the increasing demand for ambulatory care services in urology, driven by an aging population, a shortage of urologists, and evolving models of care. Adding PAs and NPs into the service delivery model for urology has led to the integration and development of care teams. How these teams function and where they function best (in the hospital or office setting) has not been studied and reported. One example of outpatient utilization involved a two-surgeon setting; employing PAs in a Canadian urology practice increased the clinical volume by 11.3 patients per day, and the yearly rate of return was a gain of USD 16,800 [45]. A reported benefit was more face time for physician/surgeon–patient contact [14]. Another advantage of having a PA in this setting was decreasing the wait time to be seen and facilitating access to urologic surgery [45].

Prompted by observations that urology PAs and NPs were increasingly taking on procedural responsibilities, a task transfer study was conducted to understand their evolving roles [46]. Using codes for the 2014–2022 national Medicare Part B beneficiary claims, the urological procedure rates performed by PAs increased by 10%, compared to a 2% increase by NPs and a 14% decrease by urologists [46]. This list included bladder ultrasounds, cystoscopy, transrectal prostate biopsies, Foley catheter placement, urodynamics testing, and more [46]. The findings were that a division of labor was underway among the three types of clinicians, and more task shifts were occurring among PAs and NPs undertaking outpatient urological procedures [46]. Skilled-clinician labor transfer or utilization in an inpatient setting was not addressed. As more urology PAs are used in urology service delivery settings, a more in-depth role delineation study of their utilization is needed to reveal areas for optimal deployment.

Historically, PAs in urology acquire their knowledge due to on-the-job training, independent study, and a collaborating physician [23]. Only 5.7% of PAs completed a postgraduate training program in 2023 after graduation from an entry-level PA program, of which 0.3% indicated urology as their area of specialty [19]. At least five academic institutions offer urology fellowships for PAs and NPs [47]. Information about professional careers following a urology postgraduate education track is needed, and future studies should explore this topic.

## 5. Limitations

As with most cross-sectional studies, there are limitations to the current study’s design and results. While the response rate of PAs for this study was 83.6%, some of the variables used in this study, such as practice attributes, burnout, job satisfaction, and annual income, are self-reported and susceptible to recall bias. Although we included all PAs who updated/confirmed their profile questions in the last 3 years, some participants may not have updated the specialty question if they have switched their specialty recently, potentially impacting the accuracy of our findings. However, the *PA Professional Profile* is a national database containing the most detailed and comprehensive information on the PA workforce.

## 6. Conclusions

In this national study, 1199 PAs indicated practicing in urology in 2022. PAs in urology were diverse in age, gender, and distribution across various employment settings but not significantly different from PAs practicing in the total of all other specialties. Most urology PAs self-identified as female, had a median age of 39, resided in urban locations, practiced in office-based private settings, worked over 40 h weekly, and offered telemedicine services for their patients. Job satisfaction was high (83.2%), whereas burnout and the intention to leave their position were relatively low for PAs in urology but comparable to PAs in all other medical disciplines. Given the predicted shortfalls of urologists and their demographic composition, this study has important implications for practice in the urology workforce that could influence workforce planning, recruitment strategies, and organizational policies specifically designed for PAs practicing in urology. Actions to consider include training PAs in low- and high-complexity procedures through additional postgraduate PA residency programs (opening more specialized urology surgical programs). Recruitment strategies that are more appealing to the younger PA workforce including work–life balance, decreasing administrative tasks, consistent training and opportunities for advancement, teamwork, mentoring to build skills and support when navigating this specialty, and a collaborative model with urologists [13].

## Figures and Tables

**Table 1:** Comparing practice characteristics of PAs in urology with PAs in all other specialties (N = 117,748).

Practice Characteristics		PAs in Urology (N = 1199)	PAs in All Other Specialties (N = 116,549)	*p*-Value ^†^
**Practice setting**	Office-based private practice	641 (53.6%)	43,019 (37.0%)	<0.001
	Hospital	434 (36.3%)	48,492 (41.7%)	
	Federal government	86 (7.2%)	5521 (4.8%)	
	Other	35 (2.9%)	19,193 (16.5%)	
**Years certified**	Mean (SD)	12.0 (8.6)	12.2 (8.8)	0.439
	Median (IQR)	11 (4–18)	10 (5–18)	
**Hours worked per week**	Up to 30	85 (7.1%)	15,679 (13.5%)	<0.001
	31–40	660 (55.0%)	66,612 (57.2%)	
	41–50	373 (31.1%)	26,963 (23.1%)	
	51+	81 (6.8%)	7239 (6.2%)	
**Patients seen each week (mean/median)**	Mean (SD)	70.2 (32.2)	67.0 (44.2)	<0.001
	Median (IQR)	65 (50–90)	60 (40–90)	
**Secondary position**	No, I work in only one clinical position	1061 (89.5%)	97,863 (84.9%)	<0.001
	Yes, I also work in a position where I do not provide direct patient care (i.e., education, research, administration)	32 (2.7%)	4459 (3.9%)	
	Yes, I work in two or more clinical PA positions	92 (7.8%)	13,009 (11.3%)	
**Participate in telemedicine**	No	574 (48.0%)	69,508 (59.9%)	<0.001
	Yes	622 (52.0%)	46,615 (40.1%)	

Abbreviations: SD = standard deviation; IQR = interquartile range. **^†^** *p*-values were calculated using Pearson Chi-Square analysis or Independent Samples Mann–Whitney U Test where appropriate.

**Table 2:** Comparing other important characteristics of PAs in urology with PAs in all other specialties (N = 117,748).

		PAs in Urology (N = 1199)	PAs in All Other Specialties (N = 116,549)	*p*-Value ^†^
**Satisfaction with present job**	Not satisfied **	195 (16.8%)	18,484 (16.4%)	0.763
	Satisfied *	969 (83.2%)	94,063 (83.6%)	
**Burnout**	No symptoms of burnout	814 (69.9%)	76,270 (67.8%)	0.124
	One or more symptoms of burnout	351 (30.1%)	36,301 (32.2%)	
**Plan to leave principal clinical position in next 12 months**	No	1098 (92.6%)	105,295 (91.3%)	0.112
	Yes	88 (7.4%)	10,067 (8.7%)	
**Plan to retire in 5 years**	No	1133 (94.7%)	109,158 (94.2%)	0.442
	Yes	63 (5.3%)	6708 (5.8%)	

* Satisfied includes “completely satisfied”, “mostly satisfied”, and “somewhat satisfied”. ** Not satisfied includes “neither satisfied nor dissatisfied”, “somewhat dissatisfied”, “mostly dissatisfied”, and “completely dissatisfied”. **^†^** *p*-values were calculated using Pearson Chi-Square analysis.

## Data Availability

The datasets presented in this article are not readily available due to the confidentiality of individualized data, but de-identified data can be available on request from the corresponding author.

## References

[1] Vespa J., Medina L., Armstrong D.M. (2020). Demographic Turning Points for the United States: Population Projections for 2020 to 2060.

[2] Administration for Community Living (2022). 2021 Profile of Older Americans.

[3] Fulmer T., Reuben D.B., Auerbach J., Fick D.M., Galambos C., Johnson K.S. (2021). Actualizing better health and health care for older adults. Health Aff..

[4] Griebling T.L., Lee A.G., Potter J.F., Harper G.M. (2021). Urology. Geriatrics for Specialists.

[5] Batmani S., Jalali R., Mohammadi M., Bokaee S. (2021). Prevalence and factors related to urinary incontinence in older adults women worldwide: A comprehensive systematic review and meta-analysis of observational studies. BMC Geriatr..

[6] Chin H.W., Kim J., Rasp G., Hristov B. (2015). Prostate Cancer in Seniors: Part 1: Epidemiology, Pathology, and Screening. Fed. Pract..

[7] Ye Z., Wang J., Xiao Y., Luo J., Xu L., Chen Z. (2024). Global burden of benign prostatic hyperplasia in males aged 60–90 years from 1990 to 2019: Results from the global burden of disease study 2019. BMC Urol..

[8] U.S. Census Bureau 2020 Census Will Help Policymakers Prepare for the Incoming Wave of Aging Boomers. US Census Bureau 2019. https://www.census.gov/library/stories/2019/12/by-2030-all-baby-boomers-will-be-age-65-or-older.html.

[9] Etzioni D.A., Liu J.H., Maggard M.A., Ko C.Y. (2003). The Aging Population and Its Impact on the Surgery Workforce. Ann. Surg..

[10] American Urological Association (AUA) (2023). The State of the Urology Workforce and Practice in the United States 2022.

[11] Nam C.S., Daignault-Newton S., Kraft K.H., Herrel L.A. (2021). Projected US Urology Workforce per Capita, 2020–2060. JAMA Netw. Open.

[12] McKibben M.J., Kirby E.W., Langston J., Raynor M.C., Nielsen M.E., Smith A.B., Wallen E.M., Woods M.E., Pruthi R.S. (2016). Projecting the Urology Workforce Over the Next 20 Years. Urology.

[13] Brand T.C., Mitchell K., Quallich S., Rubenstein J., Zwarick Shanley K., Gutierrez A., Hooper G., Motola J., Zilinskas B., Robles J. (2022). Current State of Advanced Practice Providers in Urological Practice. Urol. Pract..

[14] Langston J.P., Duszak Jr R., Orcutt V.L., Schultz H., Hornberger B., Jenkins L.C., Hemingway J., Hughes D.R., Pruthi R.S., Nielsen M.E. (2017). The Expanding Role of Advanced Practice Providers in Urologic Procedural Care. Urology.

[15] Quallich S., Lajiness S., Kovarik J., Doran T., Schultz H., Langston J.P. (2020). Standardized Office Cystoscopy Training for Advanced Practice Providers in Urology. Urol. Pract..

[16] Singh A., Lassner J.W., Sleiman M.G., Diaz A., Quallich S., Modi P.K. (2022). Advanced Practice Providers and Wait Times in Urology Offices: A Secret Shopper Study. Urol. Pract..

[17] Kapoor D.A. (2021). The Role of Advanced Practice Providers in Urology. Urol. Clin. N. Am..

[18] Boulton M.L., Beck A.J., Coronado F., Merrill J.A., Friedman C.P., Stamas G.D., Tyus N., Sellers K., Moore J., Tilson H.H. (2014). Public Health Workforce Taxonomy. Am. J. Prev. Med..

[19] National Commission on Certification of Physician Assistants (2023). 2022 Statistical Profile of Board Certified PAs by Specialty.

[20] USDA (2024). 2010 Rural-Urban Commuting Area (RUCA) Code.

[21] Rohland B.M., Kruse G.R., Rohrer J.E. (2004). Validation of a single-item measure of burnout against the Maslach Burnout Inventory among physicians. Stress Health.

[22] Dolan E.D., Mohr D., Lempa M., Joos S., Fihn S.D., Nelson K.M., Helfrich C.D. (2015). Using a Single Item to Measure Burnout in Primary Care Staff: A Psychometric Evaluation. J. Gen. Intern. Med..

[23] American Urological Association (AUA) (2020). Advanced Practice Providers for Urologic Care in the United States 2015–2019.

[24] American Urological Association Education and Research (2024). The State of Urology Workforce and Practice in the United States 2023.

[25] Dielubanza E.J., Enemchukwu E.A., Atiemo Henry H.O. (2021). Workforce Diversity in Female Pelvic Medicine and Reconstructive Surgery: An Analysis of the American Urological Association Census Data. Urology.

[26] Qin L.A., Menhaji K., Sifri Y., Hardart A., Ascher-Walsh C.J. (2023). Gender Equity in Academic Female Pelvic Medicine and Reconstructive Surgery Departments: A Cross-Sectional Observational Study. Urogynecology.

[27] Kane L. (2022). Physician Burnout & Depression Report 2022: Stress, Anxiety, and Anger.

[28] Gupta K., Tang K., Loloi J., Fang R., Meeks W., North A. (2022). Professional Burnout of Advanced Practice Providers Based on 2019 American Urological Association Census Data. Urol. Pract..

[29] Hammad T. (2023). Healthcare Workers’ Job Satisfaction. J. Res. Adm..

[30] Yilmaz F.K., Karakuş S. (2023). The relationship between healthcare workers’ satisfaction level and patients’ satisfaction: Results of a path analysis model. J. Healthc. Qual. Res..

[31] Bhatnagar K., Srivastava K. (2012). Job satisfaction in health-care organizations. Ind. Psychiatry J..

[32] Janicijevic I., Seke K., Djokovic A., Filipovic J. (2013). Healthcare workers satisfaction and patient satisfaction—Where is the linkage?. Hippokratia.

[33] Health Resources and Service Administration (2024). Physician Workforce: Projections, 2022–2037.

[34] Hooker R.S., Kuilman L., Everett C.M. (2015). Physician assistant job satisfaction: A narrative review of empirical research. J. Physician Assist. Educ..

[35] Coplan B., McCall T.C., Smith N., Gellert V.L., Essary A.C. (2018). Burnout, job satisfaction, and stress levels of PAs. JAAPA.

[36] Tetzlaff E.D., Hylton H.M., DeMora L., Ruth K., Wong Y.N. (2018). National study of burnout and career satisfaction among physician assistants in oncology: Implications for team-based care. J. Oncol. Pract..

[37] Hooker R.S., Bruza-Augatis M., Puckett K., Kozikowski A. (2024). A Comprehensive Analysis of the Physician Assistant/Associate Orthopedics Workforce. JBJS J. Orthop. Physician Assist..

[38] Bruza-Augatis M., Kozikowski A., Hooker R.S., Puckett K. (2024). Physician assistants/associates in psychiatry: A workforce analysis. Hum. Resour. Health.

[39] DePalma S.M., Hooker R.S., Bruza-Augatis M., Kozikowski A., Puckett K. (2024). Demographics and Characteristics of Cardiology Physician Assistants/Physician Associates in the United States: A Workforce Analysis. JACC Adv..

[40] McKenna R.E., Hooker R.S., Bruza-Augatis M., Puckett K., Kozikowski A. Physician Assistants in Clinical Endocrinology: Characteristics and Demographics. Endocr. Pract..

[41] Essary A.C., Bernard K.S., Coplan B., Dehn R., Forister J.G., Smith N.E., Valentin V.L. (2018). Burnout and Job and Career Satisfaction in the Physician Assistant Profession: A Review of the Literature. NAM Perspect..

[42] Lookian C., Keaton C., Keane K., Schulte E., Atchley A. (2021). Survey Analysis of Overall Job Satisfaction of Physician Assistants. J. Nurs. Interprofessional Leadersh. Qual. Saf. (JoNILQS).

[43] American Urological Association (2024). Data Sources.

[44] National Commission on Certification of Physician Assistants (2014). 2013 Statistical Profile of Certified Physician Assistants An Annual Report.

[45] Misurka J., Lajkosz K., Kenk M., Finelli A., Fleshner N.E. (2023). Quantitative and qualitative impact of physician assistants in a Canadian urology setting. Can. Urol. Assoc. J..

[46] Hooker R.S., McKenna R.E. (2024). Urology outpatient procedures by physician associates and nurse practitioners. BMC Urol..

[47] Hughes C. Advanced Practice Provider Urology Fellowships. AUA News 2023. https://auanews.net/issues/articles/2023/april-extra-2023/advanced-practice-provider-urology-fellowships.

